# The role of cognitive factors and personality traits in the perception of illusory self-motion (vection)

**DOI:** 10.3758/s13414-020-02228-3

**Published:** 2021-01-06

**Authors:** Sarah D’Amour, Laurence R. Harris, Stefan Berti, Behrang Keshavarz

**Affiliations:** 1 Centre for Vision Research, York University, Toronto, Canada; 2 Department of Psychology, York University, 4700 Keele St., Toronto, ON M3J 1P3 Canada; 3 Department of Psychology, Johannes Gutenberg University Mainz, Mainz, Germany; 4 KITE, Toronto Rehabilitation Institute–University Health Network, Toronto, Canada; 5 Department of Psychology, Ryerson University, Toronto, Canada

**Keywords:** Vection, Self-motion, Cognition, Expectation, Field dependence, Depersonalization, Anxiety, Social desirability, Sex

## Abstract

Vection is a perceptual phenomenon that describes the visually induced subjective sensation of self-motion in the absence of physical motion. Previous research has discussed the potential involvement of top-down cognitive mechanisms on vection. Here, we quantified how cognitive manipulations such as contextual information (i.e., expectation) and plausibility (i.e., chair configuration) alter vection. We also explored how individual traits such as field dependence, depersonalization, anxiety, and social desirability might be related to vection. Fifty-one healthy adults were exposed to an optic flow stimulus that consisted of horizontally moving black-and-white bars presented on three adjacent monitors to generate circular vection. Participants were divided into three groups and given experimental instructions designed to induce either strong, weak, or no expectation with regard to the intensity of vection. In addition, the configuration of the chair (rotatable or fixed) was modified during the experiment. Vection onset time, duration, and intensity were recorded. Results showed that expectation altered vection intensity, but only when the chair was in the rotatable configuration. Positive correlations for vection measures with field dependence and depersonalization, but no sex-related effects were found. Our results show that vection can be altered by cognitive factors and that individual traits can affect the perception of vection, suggesting that vection is not a purely perceptual phenomenon, but can also be affected by top-down mechanisms.

Experiencing self-motion in the absence of actual physical movement is a common phenomenon and can occur in various situations. For instance, when sitting in a stationary train ready for departure, the motion of a neighboring train can sometimes be misinterpreted as movement of one’s own train and result in the experience that one’s own train departed. Similar sensations can be observed when using Virtual Reality (VR) applications, such as VR glasses/headsets or driving/flight simulators, when users often experience the sensation of self-motion even when they remain still. The feeling of illusory self-motion is referred to as vection (Brandt, Dichgans, & Koenig, 1972; Mach, 1875). The general purpose of our study is to further enhance our knowledge of vection as a phenomenon. Moreover, since VR is constantly gaining popularity in the context of training, education, rehabilitation, and research (Adamovich, Fluet, Tunik, & Merians, 2009; Bates, 1992; Montana, Tuena, Serino, Cipresso, & Riva, 2019), a better understanding of vection may directly help to optimize VR experiences by improving the sense of realism and presence in the virtual world (Hendrix & Barfield, 1995).

Vection is a complex phenomenon with multiple contributing factors (see Hettinger, Schmidt, Jones, & Keshavarz, 2014; Palmisano, Allison, Schira, & Barry, 2015, for overviews). For instance, characteristics of the sensory information such as scene density/complexity (Brandt, Dichgans, & Koenig, 1973; Keshavarz, Philipp-Muller, Hemmerich, Riecke, & Campos, 2019; Lubeck, Bos, & Stins, 2015) or the size of the field-of-view (Allison, Howard, & Zacher, 1999; Flanagan, May, & Dobie, 2002) have been shown to affect vection. There is also evidence that vection is not entirely driven by perceptual components and that emotional states (Sasaki, Seno, Yamada, & Miura, 2012; Seno, Kawabe, Ito, & Sunaga, 2013) and cognitive factors such as contextual information or plausibility can play a modulating role (Palmisano et al., 2015; Seno et al., 2012; Wright, 2009). For instance, Riecke, Feuereissen, and Rieser (2009) reported increased auditory vection in participants who were exposed to rotating auditory sounds with their feet suspended in the air in comparison with participants whose feet touched the ground. The authors argue that the possibility of movement increased the likelihood of experiencing vection. In another study, Seno, Abe, et al. (2013) demonstrated that wearing heavy iron clogs and a weight jacket partially reduced vection as well, suggesting that the knowledge about the presence of the weights reduced the likelihood of vection to occur.

The underlying mechanisms of cognitive factors for vection remain vague. Vection is, by definition, a subjective phenomenon, and objective measures are largely missing (although some promising ideas have been recently proposed; see Berti & Keshavarz, 2020; Keshavarz, Campos, & Berti, 2015; Palmisano et al., 2015; Weech, Kenny, Calderon, & Barnett-Cowan, 2020), which makes research in this domain susceptible to reporting biases. It is possible that cognition modulates the processing of the sensory input to exert top-down effects on perceptual processes. Alternatively, cognitive processes such as expectation or knowledge about the environment might introduce a response bias, with perceptual processes remaining unaffected. In addition, it is important to determine participants’ sensitivity to cognitive factors and whether this sensitivity might vary with other factors, such as stimulus characteristics, personality, or sex. A first attempt to answer some of these questions was presented by Palmisano and Chan (2004). The authors exposed their participants to a cloud of random dots that induced linear, forward vection. Contextual information was altered by manipulating the experimental instructions and settings: One group of participants sat on a rotatable chair and was told that the purpose of the study was to investigate self-motion. The other group, in contrast, sat in a stationary chair and was told that the purpose of the study was to investigate object motion. Participants in the object-motion group were less likely to report vection (54 vs. 12 no-vection trials) and showed a significantly longer latency when they did, although no differences were found for vection duration.

The aim of the present study was to further investigate the role of cognition such as contextual factors and plausibility on the perception of vection and its interplay with (a) stimulus characteristics and (b) personality traits. In the context of stimulus characteristics, we introduced three manipulations: First, we varied the speed of the visual stimulus (slow, medium, fast) to elicit vection with varying intensity, as faster stimuli have been shown to generate stronger vection (Kennedy, Hettinger, Harm, Ordy, & Dunlap, 1996; Keshavarz, Hettinger, Vena, & Campos, 2014; So, Lo, & Ho, 2001). Second, we manipulated the participants’ expectation with regard to the possibility of actually moving by subtly varying the experimental instruction for each group: For one group, the instruction suggested that the vast majority of people experienced vection with strong intensity when exposed to this type of stimulus, whereas the instruction for the other group suggested that only very few people experienced vection and that vection was rather weak. No expectation was established for the third group (control condition). Third, we manipulated the plausibility of the laboratory setup by using a chair that was either capable of rotating around a vertical axis or that was fixed and could not rotate. Each participant watched half of the trials sitting on the chair with the rotatable setup (i.e., knowing that the chair could rotate) and the other half of the trials sitting on the chair in a fixed setup (i.e., knowing that the chair could not rotate), with the order of the setup counterbalanced between participants. We hypothesized that the manipulation of contextual information and plausibility would affect vection ratings; we expected shorter vection latencies, longer vection durations, and increased vection intensity for the high expectation group compared with the low expectation group. We expected that this effect would be particularly prevalent in the visual conditions that generated moderate vection (slow or medium speed), as stronger visual cues may dominate or override the subtle cognitive manipulations we chose. Based on previous studies (Riecke et al., 2009), we also anticipated that sitting on a chair that was known to be rotatable would increase vection compared with sitting on a fixed chair.

With respect to personality traits, we investigated four concepts that seem relevant in the context of vection: field dependence, depersonalization, anxiety, and social desirability. *Field dependence* is considered a basic cognitive style affecting perceptual processing (Boccia, Piccardi, Marco, Pizzamiglio, & Guariglia, 2016; Witkin & Goodenough, 1977) and describes the tendency to rely on external (e.g., visual) or internal (e.g., vestibular) cues with regard to perception—for instance, in the context of one’s body position with respect to gravity. That is, more field-dependent individuals rely more strongly on external cues such as reference frames, whereas less field-dependent individuals rely more strongly on internal cues such as vestibular or proprioceptive information with regard to their perception of body position. Field dependence has been shown to affect vection under certain circumstances (Keshavarz, Speck, Haycock, & Berti, 2017), but the nature of this relationship is not well understood. We hypothesized that more field-dependent participants would experience vection more easily and more intensely, because they rely more on external, visual cues than low field-dependent individuals.

*Depersonalization* describes an individual’s experience of being detached or divorced from their own body, which can result in the perception of the world as dreamlike or unreal (Mayer-Gross, 1935; Sierra & Berrios, 1998). Single episodes of depersonalization are not uncommon and are estimated to occur in approximately 20% of the population at least once in their lifetime (Aderibigbe, Bloch, & Walker, 2001), whereas chronic depersonalization is considered a dissociative disorder according to the *Diagnostic and Statistical Manual of Mental Disorders–Fifth Edition* (DSM-5) (Phillips et al., 2001). Depersonalization can affect cognitive processing: Adler et al. (2014) demonstrated that visual spatial attention differed between patients diagnosed with depersonalization disorder compared with a healthy control group. Thus, we hypothesized that depersonalization would be positively correlated with vection with those reporting higher scores of depersonalization experiencing stronger vection.

*Anxiety* is an emotion that is linked to several other phenomena, including depersonalization (Sierra, Medford, Wyatt, & David, 2012), and can be considered as a temporary emotional state or as a manifested personality trait. Interestingly, anxiety has been shown to increase the level of presence in VR (Bouchard, St-Jacques, Robillard, & Renaud, 2008), defined as the feeling of “being there” in the virtual environment (Heeter, 1992). As presence and vection are linked to each other (Prothero, 1998), it is possible that anxiety may affect vection. However, due to limited previous research, our investigation of anxiety was rather exploratory, and we had no specific hypothesis.

Lastly, *social desirability* is a type of response bias that makes participants respond in a way that they believe is viewed favorably by other (Edwards, 1957), and people with a higher social desirability could be more prone to cognitive manipulations that may affect vection. Thus, we measured the level of social desirability to explore its relationship with vection perception.

Field dependence was assessed using the rod-and-frame test (Bagust, 2005; Witkin & Asch, 1948) and depersonalization, anxiety, and social desirability were assessed by questionnaire. In addition, participants were balanced across experimental conditions according to their sex in order to investigate potential sex-related differences with respect to the sensation of vection (Darlington & Smith, 1998; Flanagan, May, & Dobie, 2005; Klosterhalfen, Pan, Kellermann, & Enck, 2006).

## Materials and methods

### Participants

A total of 67 healthy volunteers participated in this study. Seven of the participants stopped the experiment prematurely due to motion sickness and had to be removed from the study. In addition, nine participants were excluded from the data analyses due to contradicting vection ratings: For some trials, they did not report vection onset during trials, but reported increased vection intensity ratings after trial, or vice versa. Thus, the final sample size consisted of 51 participants (25 females, *M*_age_ = 23.92 years, *SD* = 7.01, age range: 18–49 years). Participants were healthy and had no self-reported recent history of stroke, active vestibular disorders, disabling musculoskeletal disorder, acute psychiatric disorder, epilepsy, and/or a diagnosis of dementia or mild cognitive impairment. Written consent was obtained prior to the experiment. The study was approved by the Research Ethics Boards of the University Health Network and York University and was designed in accordance with the Declaration of Helsinki. Participants were free to abort the experiment at any time without negative consequences and all participants were reimbursed with a $15 CDN gift card.

### Study design

To investigate the effect of contextual information on vection, participants were randomly assigned to one of three experimental groups that varied with respect to the experimental instructions. In all three groups, participants were told that the goal of this study was to investigate the subjective sensation of vection, and vection was described using the train-moving-next-to-you analogy. All participants confirmed that they were familiar with and understood the concept of vection. The manipulation of the instructions consisted of a single aspect: In one group, participants were told that the visual stimulus that they were about to see generated vection in 82% of all observers in previous studies, and for those who experienced it, the sensation was very strong and compelling as indicated by high intensity ratings (“strong expectation group”). In the second group, participants were told that the visual stimulus that they were about to see generated vection in 18% of observers in previous studies and for those who experienced it, the sensation was very weak and not compelling as indicated by low intensity ratings (“weak expectation group”). The remainder of the instruction was identical. The third group acted as a control condition, and no percentage score or description of vection sensation strength was given (“control group”).

To investigate the effect of plausibility on vection, the rotatability of the experimental chair was modified as a within-subjects factor: a custom-made wooden clamp was attached to lower part of the chair that prevented the chair from rotating and kept it stationary during half of the trials. In other words, participants were exposed to the moving stimuli sitting either on a chair that they knew was capable of rotating or sitting on the same chair that they knew was fixed. The rotatability of the chair was demonstrated to the participants immediately before they sat on it. The order of the chair configuration was counterbalanced.

Finally, to manipulate vection intensity, we chose three different stimulus speeds: slow (0.5 cycles per s), medium (1 cycle per s), and fast (2 cycles per s). A cycle was defined as a combination of a single black and white bar with a total of 134 pixels (or 5.16°) per cycle. Consequently, the final design resulted in a 3 × 2 × 3 design, including the between-subjects factor expectation (weak, strong, no expectation) as well as the within-subjects factors chair configuration (rotatable, fixed) and stimulus speed (slow, medium, fast).

### Stimuli and apparatus

**Fig. 1:**
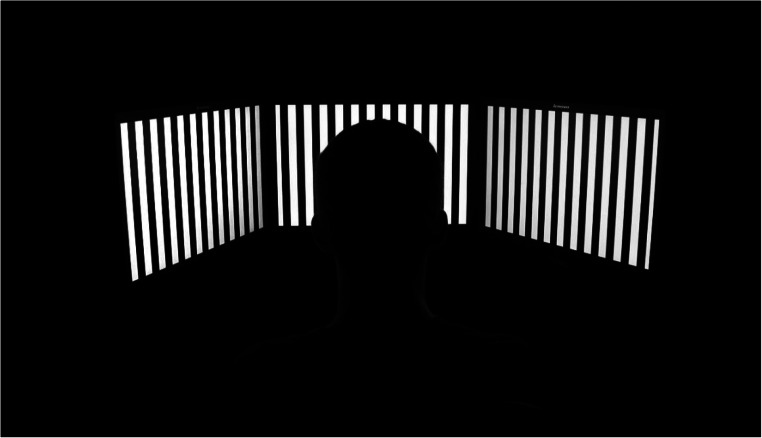
Experimental setup and stimuli

The stimuli consisted of a pattern of alternating black and white vertical stripes that moved horizontally either to the right or left (Fig. 1). The spatial frequency of the stimulus was 0.19 cycles/degree. A red fixation cross was presented in the center of the middle screen. The stimuli were presented on an array of three 24-in. Lenovo (ThinkVision) monitors that were aligned next to each other (approx. angle of 120° between monitors). The refresh rate was 60 Hz and the display resolution was set to 1,920 × 1,200 pixels for each monitor. Participants were seated in a height-adjustable rotatable chair with their eye-height adjusted to the monitors’ center. Participant were seated 32 cm in front of the screen, resulting in a field of view (FOV) of approximately 228 degrees horizontally and 48 degrees vertically. This setup was demonstrated to reliably trigger vection in previous studies (Keshavarz et al., 2017).

The stimuli varied in speed (slow, medium, fast) and direction (right, left), resulting in six stimulus combinations. The duration of each trial was 60 s and consisted of a 2-s static phase, 3-s acceleration phase, 45 s of constant motion, 3-s deceleration phase, and a 7-s static phase. Between the trials, the bars disappeared and the screen turned black. Each trial was repeated three times for each condition, and all participants watched all 18 trials, once sitting on the experimental chair that they knew could rotate, and once where they knew it could not, resulting in a total of 36 trials that were run in four blocks of nine trials each. The two blocks that belonged to the same chair configuration were run together and were counterbalanced across participants.

### Dependent measures

#### Vection measures

Vection was measured using three common metrics (see Berti & Keshavarz, 2020; Palmisano et al., 2015): vection onset time, vection duration, and vection intensity. With this, we can explore different aspects of vection processing such as temporal aspects and the subjective experience (for details, see Seno et al., 2017). Onset time was defined as the time that elapsed before vection was first reported. Vection duration was defined as the total time that participants experienced vection during each trial. Vection onset time and duration were recorded using a button press. Participants were asked to press the left button of a computer mouse as soon as they first experienced vection and were asked to keep the button pressed as long as vection lasted. When the sensation of vection disappeared, they were asked to release the button. Since vection is not a stable percept and multiple phases of vection can be experienced within a single trial, participants were asked to repeat this procedure throughout the duration of the trial as necessary. The very first button press indicated vection onset time, and the total duration of vection per trial was calculated using the sum of all times when participants hold the button pressed. Vection intensity was measured after each trial using a verbally reported rating scale ranging from 0 (*no vection at all*) to 10 (*intense vection*). Participants’ were also asked at the end of each trial to report the direction of vection (left or right) that they experienced.

#### Visually induced motion sickness

To control for potential adverse side-effects associated with vection such as visually induced motion sickness (VIMS; Keshavarz, Riecke, Hettinger, & Campos, 2015), the Fast Motion Sickness Scale (FMS; Keshavarz & Hecht, 2011)—a subjective rating scale ranging from 0 (*no sickness*) to 20 (*severe sickness*)—was administered after each trial. To ensure participants’ well-being during the experiment and to ensure that no severe cases of nausea occurred, VIMS was measured throughout the experiment. However, no statistical analysis were done on VIMS as this was not the focus of our study.

### Personality trait measures

At the end of the experiment, a series of baseline measures were taken: field dependence, anxiety, depersonalization, and social desirability.

#### Field dependence

Participants’ level of field dependence was measured using a computerized version of the rod-and-frame test (CRAF; Bagust, Rix, & Hurst, 2005). The CRAF consists of five linearly aligned dots (i.e., the rod) surrounded by a luminescent frame. Participants were asked to change the rod’s position to vertical using the buttons of a mouse to align it with gravity. The frame surrounding the rod was either tilted clockwise or counterclockwise (18°), remained stable, or was not visible at all. Deviations in the participants’ alignment from vertical (or “errors”) were measured in degrees and used as a measure of field dependence. The CRAF was presented on a large projection screen (300 cm × 196 cm). The frame size was 187 × 187 cm, corresponding to 50.1° horizontally and vertically. Participants were asked to wear a pair of goggles (no lens strength) with customized facing that limited the visible visual field to the projection screen. In each of the four frame conditions (no frame, not tilted, tilted clockwise, tilted counterclockwise), the rod was adjusted four times. An averaged CRAF score (deviation from true vertical in degrees: average value of [(Frame −18°) − (Frame 0°)] and [(Frame +18°) − (Frame 0°)]) was calculated and used for the statistical analyses.

#### Anxiety

The State-Trait Anxiety Inventory (STAI) for Adults (Spielberger & Sydeman, 1994) is a 40-item self-report questionnaire that was developed to measure the presence and severity of anxiety symptoms using two subscales (20-items each) to evaluate both state and trait anxiety (internal consistency coefficients = .86 to .95). State anxiety records the current state of anxiety (how participants feel “right now”; S-Anxiety scale), whereas trait anxiety focuses on the stable aspects of anxiety (how participants feel “generally”; T-Anxiety scale). Each question uses a scale from 1 (S-Anxiety scale*: not at all*; T-Anxiety scale: *almost never*) to 4 (S-Anxiety scale: *very much so*; T-Anxiety Scale: *almost always*). Scores are added together, with reverse scoring of anxiety-absent questions (19 out of 40 questions), resulting in subscale scores ranging from 20 to 80, with lower scores indicating lower levels of anxiety and higher scores indicating higher levels of anxiety. For the purpose of the present study, we focused exclusively on trait anxiety.

#### Depersonalization

The Cambridge Depersonalization Scale (CDS; Sierra & Berrios, 2000) is a 29-item self-report questionnaire that evaluates whether specific but presumably strange experiences occurred over that past 6 months (Cronbach alpha = .89 and split-half reliability = .92). The experiences are specifically described and evolve around the feeling of detachment from the own body. For instance, item 24 asks about whether the following sensation occurs: “When I move, it doesn’t feel as if I were in charge of the movements, so that I feel ‘automatic’ and mechanical as if I were a ‘robot.’” Participants were then asked to indicate the frequency of this sensation on a scale from 0 (*never*) to 4 (*all the time*) and the duration on a 1 (few seconds) to 6 (*more than a week*) scale. Scores were summed to obtain total frequency and total duration scores, which were then added together to obtain a Global Depersonalization Score, ranging from 0 to 290.

#### Social desirability

Participants’ level of social desirability was measured using the Marlowe-Crowne Social Desirability Scale (SDS; Crowne & Marlowe, 2011). The SDS is a 33-item long self-report questionnaire that inquires the participants’ general behavior and sentiments in certain situations (e.g., “I like to gossip at times,” or “I have never intensely disliked anyone.”). Participants respond with *true* or *false* to each question. The number of *true* responses is then added up to calculate the SDS total score. Higher scores on the SDS suggest that a person is more likely to respond in a way that seeks social approval.

### Procedure

Participants provided written consent prior to the experiment. A prescreening of participants’ health was conducted to ensure participant eligibility for the study. No participant had to be excluded based on the prescreening. Participants were then pseudorandomly assigned to one of the three experimental groups (strong expectation group, weak expectation group, no expectation group). They were given the respective written instruction. Before participants were seated, the experimenter demonstrated that the chair could or could not actually rotate, depending on the experimental chair condition. A short practice session with two trials was used to familiarize participants with the experimental procedure. Halfway through the experiment (i.e., after the first two blocks of trials), the chair configuration was changed by attaching or removing the customized wooden clamp from underneath the chair seat to enable or disable chair rotatability before continuing with the study. The order of chair configuration was counterbalanced and the order of trials within each block was randomized. After stimulus presentation was complete, participants filled out the anxiety and depersonalization questionnaires, and completed the CRAF before being debriefed.

### Data analysis

For all statistical analyses, the Statistical Package for Social Sciences (SPSS, IBM, Version 26) as well as the statistical software R were used. A priori significance was set to alpha = .05 and post hoc multiple comparisons were performed using Bonferroni corrections. Partial eta square (η_p_^2^) was calculated as effect size.

Eleven of the 51 participants provided inconsistent vection ratings for some of the trials (fewer than two trials in total per participant): They did not report vection onset, but reported increased vection intensity ratings after the trial, or vice versa. These trials were removed from the data analysis (total of 15 trials). To analyze vection onset time, the time of the first button press after the visual stimulus started to move (i.e., after the 2-s static period) was used as the onset of vection. If the first button press occurred before stimulus motion, it was not considered as onset time—instead, the subsequent button press was used. Due to technical issues, two participants could not perform the CRAF and had to be removed from the statistical analyses involving field dependence.

One-way analyses of variance (ANOVAs) including the factor experimental group (weak expectation, strong expectation, control group) were calculated for the personality trait measures social desirability, field dependence, anxiety, and depersonalization to control for group differences. No significant differences were found in any of the personality trait measures (*p*s > .668), indicating that the three groups did not differ with respect to these personality traits (see Table 1).

**Table 1:** Mean (*SD*) scores for personality trait measures separated by experimental condition

	Experimental condition (expectation)		
	Control group	Weak expectation	Strong expectation
STAI	40.81 (9.61)	40.21 (9.28)	39.56 (11.54)
CDS	33.62 (24.98)	40.37 (37.53)	38.69 (43.66)
CRAF	1.43 (2.25)	1.82 (2.72)	2.28 (4.29)
SDS	18.56 (3.95)	18.05 (3.89)	19.37 (5.14)

*Note*. STAI = State-Trait Anxiety Inventory; CDS = Cambridge Depersonalization Scale; CRAF = Computerized Rod and Frame test; SDS = Social Desirability Scale

## Results

### Number of non-vection trials

A summary of the number and percentage of trials that did not generate vection is given in Table 2. A mixed repeated-measures analysis of variance (rmANOVA) including the within-subjects factors chair configuration (rotatable, fixed) and stimulus speed (slow, medium, fast) and the between-subjects factor expectation (weak, strong, no expectation) was calculated for the number of non-vection trials. Results showed a main effect of speed, *F*(2, 96) = 16.52, *p* < .001, η_p_^2^ = .256, indicating that the number of non-vection trials increased as the stimulus speed decreased. A main effect of chair *F*(1, 96) = 6.90, *p* = .012, η_p_^2^ = .126, showed that the number of non-vection trials was higher in the rotatable chair condition (23.7%) compared with the fixed chair condition (17.6%). No other main effects or interactions were found.

**Table 2:** Number (percentage) of non-vection trials separated by stimulus speed, expectation, and chair configuration

Expectation	Chair	Speed			Total
		Fast	Medium	Slow	
Control (*N* = 16)	Fixed	7 (14.6%)	9 (18.8%)	19 (39.6%)	35 (24.3%)
	Rotatable	7 (14.6%)	10 (29.8%)	25 (52.1%)	42 (29.2%)
Weak (*N* = 19)	Fixed	2 (3.5%))	10 (17.5%)	19 (33.3%)	31 (18.1%)
	Rotatable	8 (14.0%)	8 (14.9%)	27 (47.4%)	43 (25.2%)
Strong (*N* = 16)	Fixed	0 (0.0%)	5 (10.4%)	11 (22.9%)	16 (11.1%)
	Rotatable	1 (2.1%)	5 (10.4%)	18 (37.5%)	24 (16.7%)
Total (*N* = 51)	Fixed	9 (5.9%)	24 (15.7%)	49 (32.0%)	82 (17.6%)
	Rotatable	16 (10.5%)	23 (15.0%)	70 (45.8%)	109 (23.7%)

### Expectation and chair configuration

Mixed rmANOVAs including the within-subjects factors stimulus speed (slow, medium, fast) and chair configuration (rotatable, fixed) and the between-subjects factor expectation (weak, strong, no expectation) were computed for the vection measures of onset time, duration, and intensity. Sex (male, female) was added as another between-subjects factor to test for differences between females and males with respect to vection.

#### Vection onset time

**Fig. 2:**
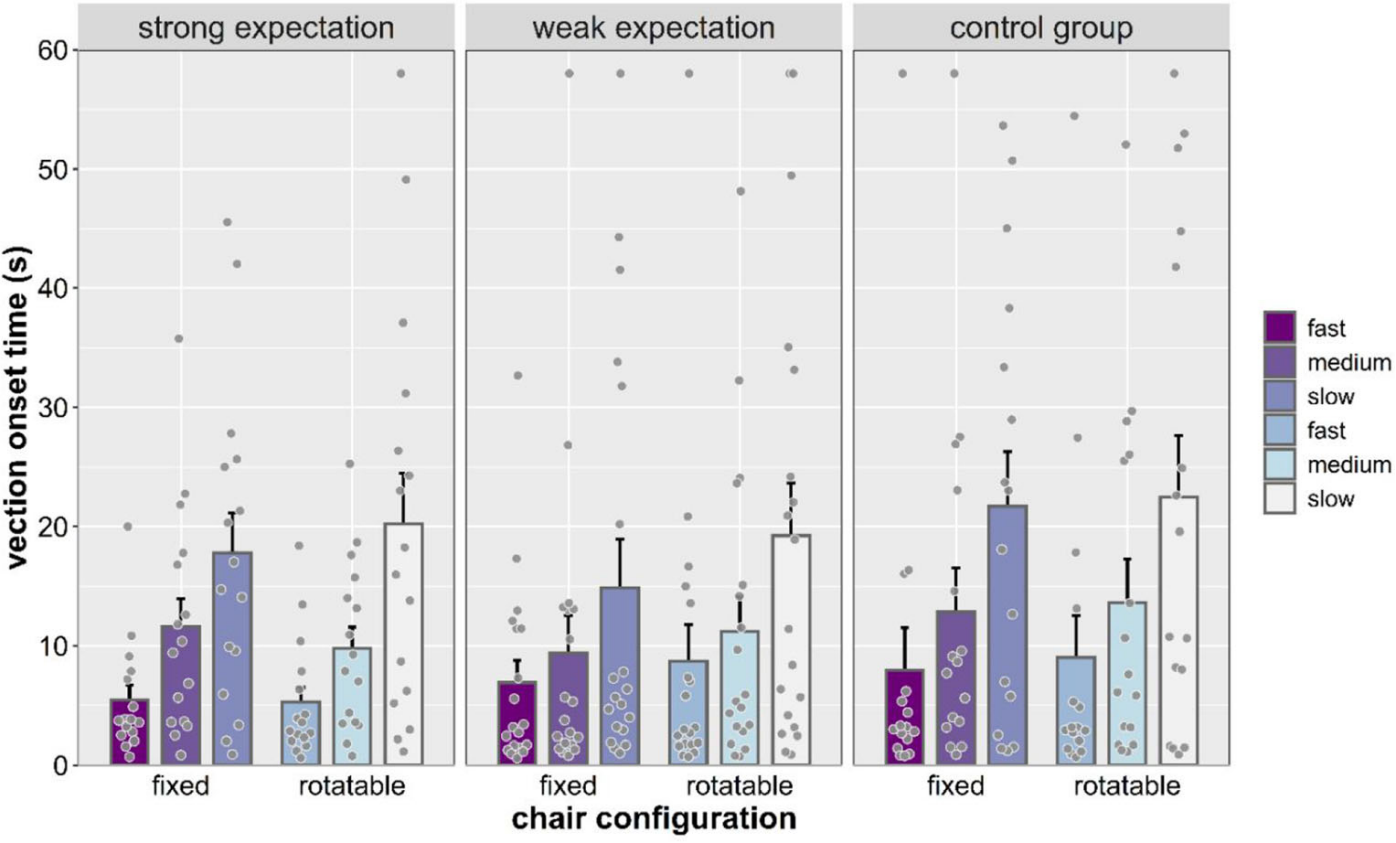
Vection onset time (s) separated by expectation, chair configuration, and stimulus speed. Individual dots represent means for each participant. Error bars represent *SEM*

To account for the trials that did not induce vection (see Table 1), we used the maximum duration of the trial (58 s) as their “onset time” for the data analysis (see Keshavarz et al., 2014; Palmisano & Chan, 2004). A significant main effect of speed was found, *F*(2, 90) = 37.685, *p* < .001, η_p_^2^ = .456, revealing that vection onset times were reduced as stimulus speed increased (all *p*s < .001; see Fig. 2). Main effects of chair configuration, *F*(1, 45) = 3.222, *p* = .079, η_p_^2^ = .067, expectation, *F*(2, 45) = 0.245, *p* = .784, η_p_^2^ = .011, and sex, *F*(1, 45) = 3.393, *p* = .072, η_p_^2^ = .070, were not significant. No significant interaction was found.

#### Vection duration

**Fig 3:**
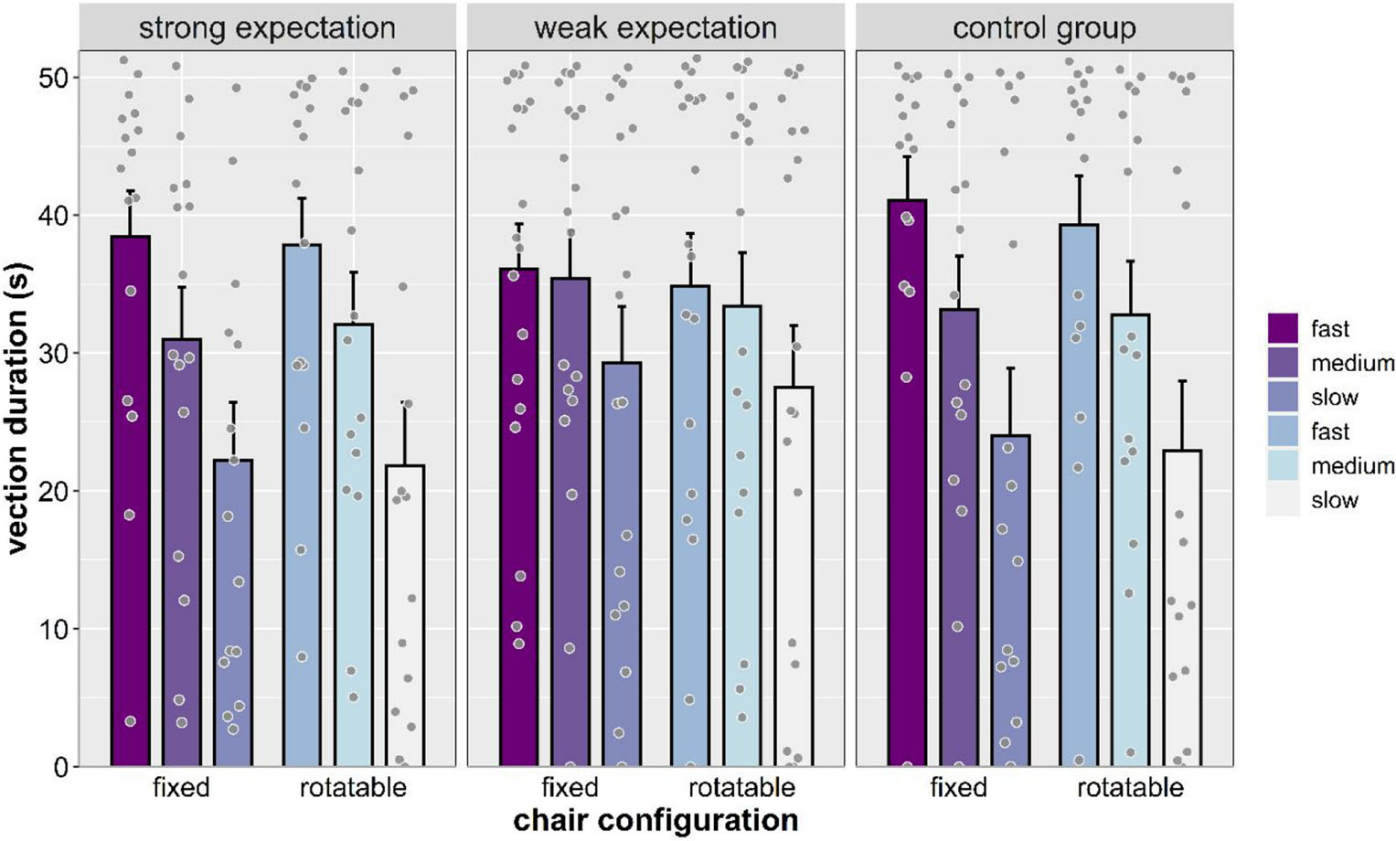
Vection duration (s) separated by expectation, chair configuration, and stimulus speed. Individual dots represent means for each participant. Error bars represent *SEM*

A significant main effect of speed was found, *F*(2, 90) = 46.296, *p* < .001, η_p_^2^ = .507, revealing that vection duration increased as stimulus speed increased (all *p*s < .001; see Fig. 3). Main effects of chair configuration, *F*(1, 45) = 2.199, *p* = .145, η_p_^2^ = .047, expectation, *F*(2, 45) = 0.128, *p* = .880, η_p_^2^ = .006, and sex, *F*(1, 45) = 0.425, *p* = .518, η_p_^2^ = .009, were not significant. No significant interaction was found.

#### Vection intensity

**Fig. 4:**
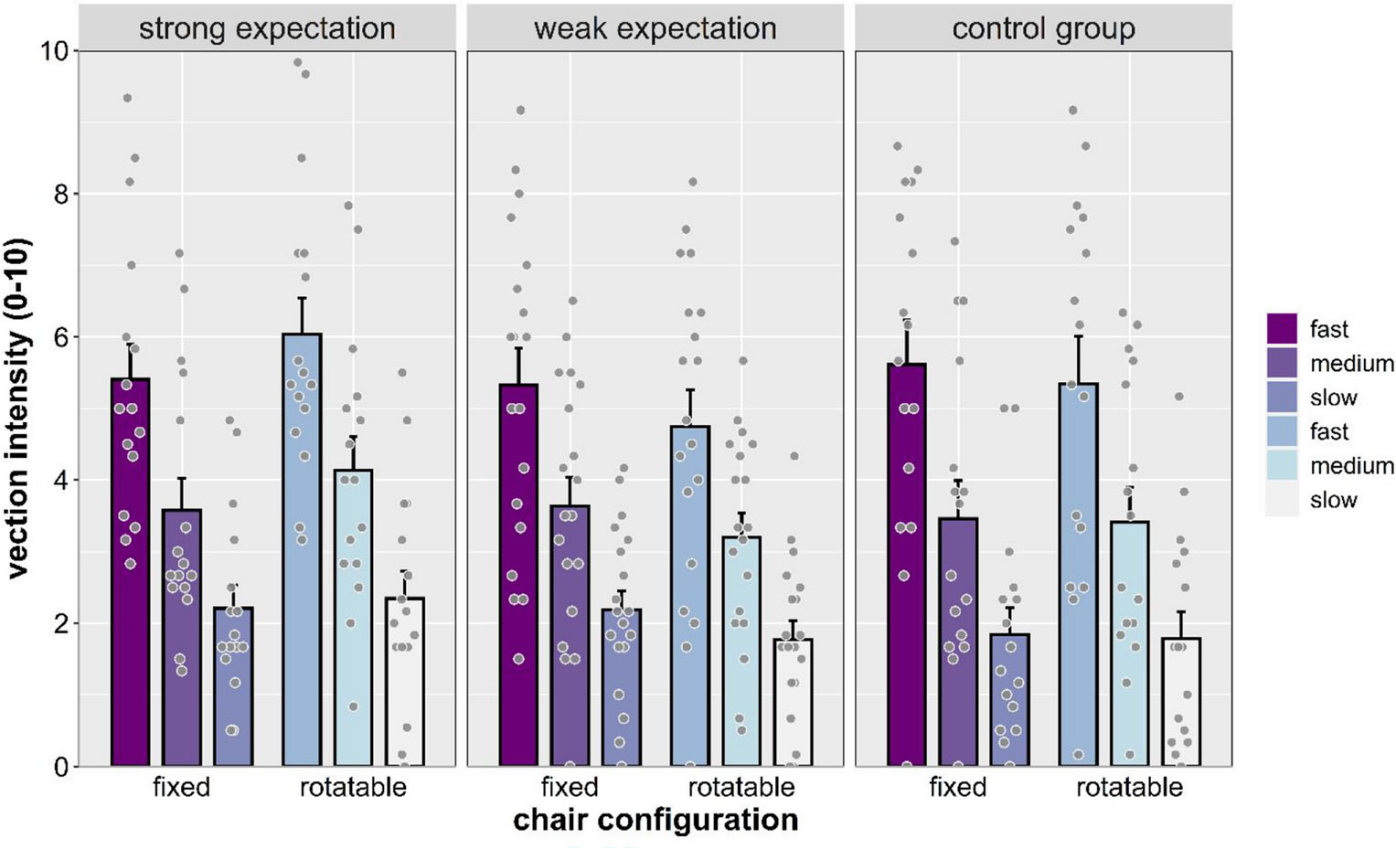
Vection intensity separated by expectation, chair configuration, and stimulus speed. Individual dots represent means for each participant. Error bars represent *SEM*

**Fig. 5:**
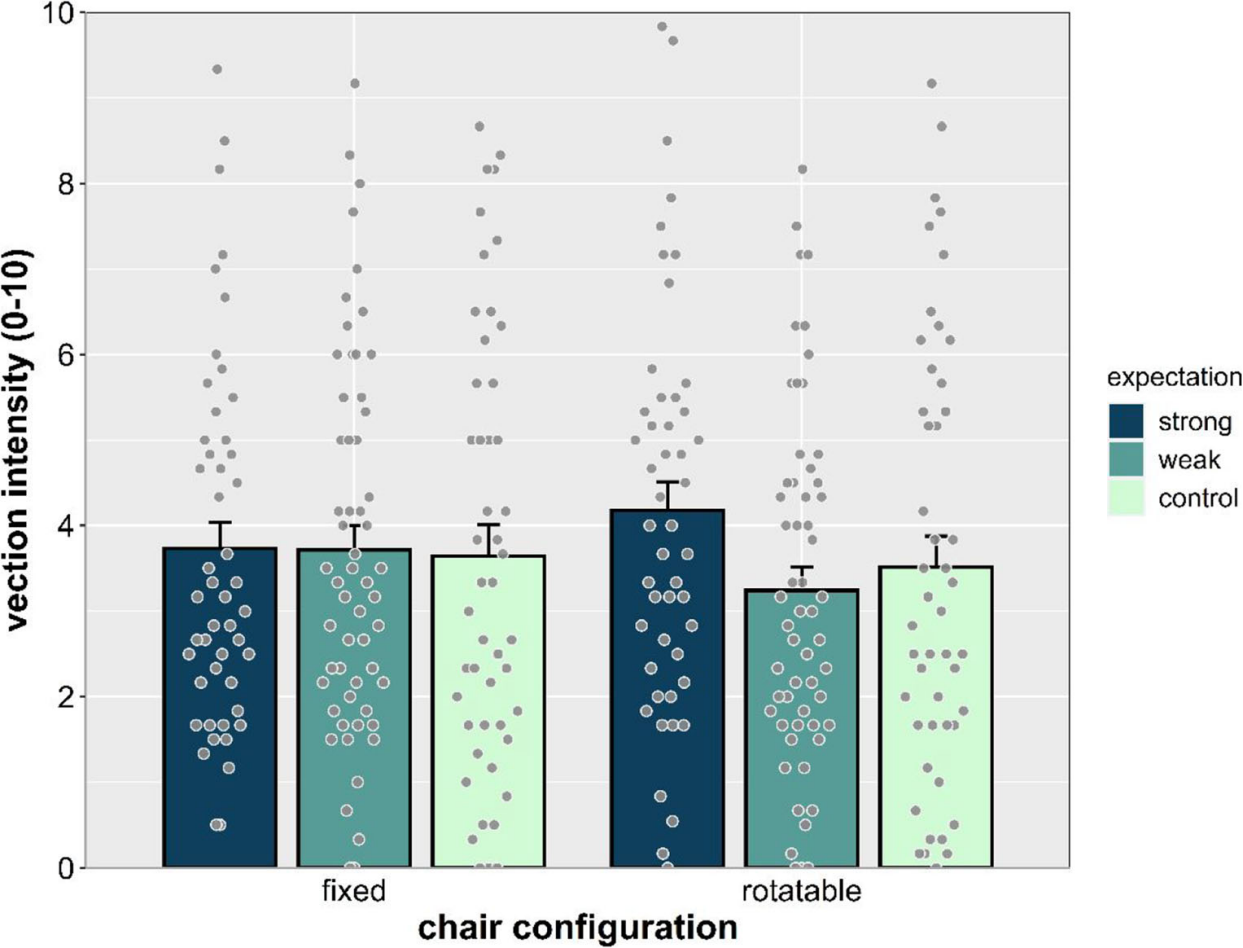
Vection intensity separated by chair configuration and expectation. Individual dots represent means for each participant. Averaged across stimulus speed. Error bars represent *SEM*

A significant main effect of speed was found, *F*(2, 90) = 170.340, *p* < .001, η_p_^2^ = .791, showing that vection intensity ratings increased with speed (all *p*s < .001; see Fig. 4). Main effects of chair configuration, *F*(1, 45) = 0.177, *p* = .685, η_p_^2^ = .004, expectation, *F*(2, 45) = 0.371, *p* = .692, η_p_^2^ = .016, and sex, *F*(1, 45) = 0.020, *p* = .888, η_p_^2^ = .020, were not significant. A significant interaction between chair configuration and expectation was found, *F*(2, 45) = 5.640, *p* = .009, η_p_^2^ = .191, indicating that expectation affected vection intensity when the chair was in rotatable configuration, but not in the fixed configuration (see Fig. 5). Follow-up post hoc comparisons showed significant differences between the rotatable and fixed chair conditions in the weak expectation group (*p* = .039) and a nonsignificant trend in the strong expectation group (*p* = .061). No other interaction was significant.

### Field dependence, depersonalization, anxiety, and social desirability

Pearson correlations between the between the CRAF score, CDS score, STAI trait score, and SDS score with all vection measures were calculated to investigate the role of field dependence, depersonalization, anxiety, and social desirability on vection (see Table 3). Moderate, positive correlations were found between the CRAF score and vection duration across all experimental conditions. CRAF also correlated moderately with vection intensity, but only when the stimulus was moving slowly. No significant correlations were found with vection onset time. For depersonalization, moderate positive correlations were found with regard to vection intensity across all experimental conditions. No significant correlations were found for anxiety or social desirability with any of the vection measures.

**Table 3:** Pearson correlations between the CRAF score, CDS score, STAI trait score, and the SDS score with all vection measures, separated by chair configuration and stimulus speed

Score	Vection measure	Fixed chair			Rotatable chair		
		Fast	Medium	Slow	Fast	Medium	Slow
CRAF (*df* = 47)	Onset time	−.187	−.180	−.278	−.163	−.225	−.281
	Duration	.321*	.356*	.383**	.346*	.378**	.406**
	Intensity	.179	.189	.307*	.198	.192	.287*
CDS (*df* = 49)	Onset time	−.082	−.071	−.149	−.051	−.074	−.095
	Duration	.220	.186	.179	.126	.130	.160
	Intensity	.297^*^	.307^*^	.406^**^	.297^*^	.368^**^	.381^**^
STAI (*df* = 49)	Onset time	−.021	.001	.046	.031	.081	.050
	Duration	−.151	−.167	−.118	−.225	−.239	−.145
	Intensity	−.081	−.050	.100	−.175	−.049	−.017
SDS (*df* = 49)	Onset time	−.149	−.116	−.025	−.128	−.158	−.014
	Duration	.039	.016	−.097	.082	.094	−.065
	Intensity	−.031	.056	−.112	.064	−.005	−.070

**p < .01, *p < .05

To investigate the role of field dependence on vection more closely, a median split of the averaged CRAF scores (median = 0.755) was used to separate participants into two subgroups: a low-field-dependent group (low CRAF scores; n = 25, *M* = 0.03, *SD* = 0.58; 18 males, seven females) and a high-field-dependent group (high CRAF scores; *n* = 24, *M* = 3.71, *SD* = 3.55; eight males, 16 females; see Keshavarz et al., 2017). This new factor field dependence (high, low) was added to the rmANOVA as an additional between-subjects factor for all vection measures.

The main findings were identical to the rmANOVA described previously. In addition, a significant main effect of field dependence was revealed for vection onset times, *F*(1, 37) = 5.274, *p* = .027, η_p_^2^ = .125, vection duration, *F*(1, 37) = 11.257, *p* = .002, η_p_^2^ = .233, and vection intensity, *F*(1, 37) = 6.093, *p* = .018, η_p_^2^ = .141. High field-dependent participants reported shorter vection onset times, longer vection duration, and more intense vection compared with low field-dependent participants (see Table 4).

**Table 4:** Mean (*SD*) scores for all vection measures collapsed across stimulus speed and separated by field dependence

Field dependence	Vection measure		
	Onset time (s)	Duration (%)	Intensity (0–10)
High	8.24 (8.00)	38.91 (10.64)	4.14 (1.51)
Low	17.30 (14.15)	24.45 (15.00)	3.19 1.65)

## Discussion

The aim of the present study was to investigate the role of cognitive factors and individual traits on the perception of vection. Our results indicate that indeed cognitive factors can alter the perception of vection. We found that expectation affected vection intensity, but only when the chair was known to be rotatable. Interestingly, while the effect of expectation was limited to the perceived intensity of vection, variation in the sensory input (i.e., the speed of visual motion stimuli) affected all three vection measures: onset time, intensity, and duration. Field dependence and depersonalization were also found to modulate the experience of vection, but again, not every aspect of vection was correlated with these traits. This pattern of results demonstrates that top-down effects on vection exist and reveals a complex interplay between contextual effects and perceptual factors.

### Cognitive factors in the context of vection

The results of the present study suggest that cognitive factors can affect the perception of vection, but only under certain contextual settings. That is, our manipulation of the experimental instruction successfully altered vection intensity when the chair was in a rotatable configuration, but not when the chair was fixed. With regard to this finding, two aspects are worth noting: First, we applied only a rather modest manipulation of participants’ expectations. Second, variation of the context (i.e., the rotatability of the chair) was introduced without explicit reference to self-motion. Our top-down effects, therefore, imply the processing of different sources of information about the likelihood of self-motion and the integration of this contextual information into the potential self-motion perception triggered by visual motion. Importantly, this is supported by other studies suggesting cognitive effects on vection, because all studies applied context information that was not directly related to the self-motion, but only “informed” the participants indirectly about the likelihood of self-motion (for instance, wearing additional weights, which made self-motion more unlikely; Seno, Abe, et al., 2013). Interestingly, our instructions only affected vection intensity, but not vection onset time or duration, suggesting that vection onset time and duration are subject to different processes than perceived vection intensity. Although these three parameters are typically strongly correlated with each other, they may indeed represent different aspects of vection (for details see Seno et al., 2017). Our results suggest that cognitive factors may influence the intensity of a vection sensation, but leave its temporal aspects (onset, duration) unaffected.

Our findings are in accordance with previous work suggesting that vection can be modulated by cognitive factors such as contextual information and plausibility. For instance, Riecke, Schulte-Pelkum, Avraamides, Heyde, and Bülthoff (2006) exposed participants to a rotating visual stimulus that either consisted of a photograph of a street scene or of a scrambled version of the same photograph. Their results suggested that ecologically valid photographs generated more vection compared with the scrambled stimulus, although visual components such as contrast, color, speed, and spatial frequency content remained constant. In addition, a study by Mursic, Riecke, Apthorp, and Palmisano (2017) exposed their blindfolded participants to musical stimuli (a Shepard-Risset glissando) that provided metaphorical auditory motion stimulation. That is, the musical stimuli did not deliver any spatialized cues, but rather conveyed a descent or ascent in pitch. The music alone successfully created vertical vection in most of their participants. With regard to plausibility, Wright (2009) demonstrated that vection was more compelling when the laboratory settings allowed the possibility of movement (e.g., participants sitting on top of an oscillator vs. sitting on a fixed chair). Interestingly, as in our study, vection latency was not affected by this cognitive manipulation. The authors conclude that some, but not all, aspects of vection can be modulated by contextual factors. Similar results were found by Riecke et al. (2009), who demonstrated that participants having their feet suspended in the air reported stronger auditory vection compared with those with their feet on the ground.

From a computational point of view, a pure processing of sensory information in order to adapt to a moving environment seems adequate, and the question arises as to why an elaborate processing of contextual information that modulates the perceptual response should exist. We cannot give a complete answer to this question on basis of this study, but we speculate that processing of information relying on different sources of motion related-information (including likelihood information) is more flexible and can more reliably predict potential changes in the environment. Take, for example, a surfer on a surfboard: By processing and extrapolating the motion of an approaching yet distant wave, she can estimate the point in time at which the board (and she) will start to move, and when to apply some strokes to catch the wave; in this context, a pure bottom-up processing of self-motion would be too late to allow for preparation of the upcoming motion (and she will miss the perfect wave!).

The perception of vection undoubtedly comes about from the sufficient activation of multisensory cortical areas normally involved in processing all self-motion related cues (Chen, DeAngelis, & Angelaki, 2011; Kleinschmidt et al., 2002). Organisms clearly need to have cognitive awareness of their self-motion and there are extensive reciprocal connections between the frontal cortical areas traditionally associated with higher cognitive functioning including navigation and decision-making, and areas known to be involved in self-motion processing, such as the posterior parietal cortex (Battaglia-Mayer, Caminiti, Lacquaniti, & Zago, 2003; Gu, Cheng, Yang, Deangelis, & Angelaki, 2016) and areas that are stimulated by large-field visual stimuli (Avila, Lakshminarasimhan, DeAngelis, & Angelaki, 2019; Bremmer, Klam, Duhamel, Ben Hamed, & Graf, 2002). These connections may be the basis by which self-motion can inform higher cognitive functioning, such as decision-making, and also through which expectations might modulate self-motion perception as demonstrated in this study (see also Berti & Keshavarz, 2020).

### Exploration of personality trait effects and sex on vection

The relationship between individual traits and vection has only been rarely investigated in the past. Seno, Yamada, and Ihaya (2011) explicitly investigated how personality factors may affect the perception of vection, and the authors found evidence that vection was weaker in participants with a higher level of narcissism, whereas other Big Five personality traits (openness, conscientiousness, extraversion, agreeableness) showed no correlation with vection measures.

The present study explored the relationship between field dependence, depersonalization, anxiety, and social desirability on vection and revealed interesting findings. For instance, high field-dependent participants reported shorter vection onset times, longer vection duration, and more intense vection compared with their low field-dependent counterparts. In other words, participants who typically rely more strongly on surrounding visual cues than others are more likely to experience more prolonged and more intense vection. This result seems plausible, as the only visible visual cues provided during the experiment consisted of the horizontally moving bars. Thus, it may not be surprising that individuals who rely more on visual cues with regard to their body position in space experience stronger vection than those who rely more on internal (e.g., vestibular and proprioceptive) cues. This finding is at least partially in accord with our previous work (Keshavarz et al., 2017) where a difference in vection measures between high and low field-dependent participants was found for some, but not all, experimental settings.

With respect to depersonalization, we found moderate, positive correlations with vection intensity, suggesting that participants who report higher susceptibility to “out of the body” experiences are more likely to experience stronger vection. Vection is an illusion of self-motion operating through shared vestibular pathways, and artificial vestibular stimulation and vestibular disease have been shown to increase the likelihood of depersonalization experiences (Jáuregui Renaud, 2015; Lopez & Elzière, 2018; Yen Pik Sang, 2006). The comparatively strong correlation between vection intensity and the depersonalization score in our slow-speed conditions suggests that people experiencing depersonalization might be more vulnerable to motion-related visual stimulation. Vection susceptibility may provide a useful tool with which to further explore the cognitive basis of depersonalization and out-of-body experiences. Interestingly, since anxiety did not affect vection in our study despite depersonalization and anxiety often being correlated (Sierra et al., 2012), we can conclude that we tapped a specific relationship between depersonalization and vection that is not moderated by anxiety.

We found no effect of social desirability on vection, which also has previously been shown not to affect susceptibility to motion sickness (Hemmerich, Shahal, & Hecht, 2019). In fact, this is a promising finding for vection research in general, as it suggests that the level of vection reported during experimental research is likely not affected by reporting biases due to social desirability. Previous work suggested that the presence of an audience may alter the perception of vection (Seno, 2013), suggesting a social component. However, it seems unlikely that the presence of other people causes differences in vection ratings due to social desirability. Seno’s (2013) finding is probably better explained by distraction.

Finally, no differences were found between males and females in any of the vection ratings, suggesting that sex is not a crucial factor for the perception of vection. This finding supports previous research that found sex-related differences for visually induced motion sickness but not for vection (Flanagan et al., 2005; Klosterhalfen et al., 2006).

## Conclusions

We have shown that high-level cognitive factors and individual traits such as field dependence and depersonalization can affect the perception of vection, confirming that vection can no longer be treated as a purely perceptual phenomenon. Instead, our results expand our emerging understanding of the two-way influences in which cognition is informed by self-motion and vestibular information, and high-level cognitive factors in turn influence self-motion perception.

## References

[CR1] Adamovich SV, Fluet GG, Tunik E, Merians AS (2009). Sensorimotor training in virtual reality: A review. NeuroRehabilitation.

[CR2] Aderibigbe YA, Bloch RM, Walker WR (2001). Prevalence of depersonalization and derealization experiences in a rural population. Social Psychiatry and Psychiatric Epidemiology.

[CR3] Adler J, Beutel ME, Knebel A, Berti S, Unterrainer J, Michal M (2014). Altered orientation of spatial attention in depersonalization disorder. Psychiatry Research.

[CR4] Allison RS, Howard IP, Zacher JE (1999). Effect of field size, head motion, and rotational velocity on roll vection and illusory self-tilt in a tumbling room. Perception.

[CR5] Avila E, Lakshminarasimhan KJ, DeAngelis GC, Angelaki DE (2019). Visual and vestibular selectivity for self-motion in macaque posterior parietal area 7a. Cerebral Cortex.

[CR6] Bagust J (2005). Assessment of verticality perception by a rod-and-frame test: Preliminary observations on the use of a computer monitor and video eye glasses. Archives of Physical Medicine and Rehabilitation.

[CR7] Bagust J, Rix GD, Hurst HC (2005). Use of a Computer Rod and Frame (CRAF) Test to assess errors in the perception of visual vertical in a clinical setting—A pilot study. Clinical Chiropractic.

[CR8] Bates J (1992). Virtual reality, art, and entertainment. Presence: Teleoperators and Virtual Environments.

[CR9] Battaglia-Mayer A, Caminiti R, Lacquaniti F, Zago M (2003). Multiple levels of representation of reaching in the parieto-frontal network. Cerebral Cortex.

[CR10] Berti, S., & Keshavarz, B. (2020). Neuropsychological approaches to visually-induced vection: An overview and evaluation of neuroimaging and neurophysiological studies. *Multisensory Research*, 1–34. Advance online publication. 10.1163/22134808-bja10035 10.1163/22134808-bja10035 33706273

[CR11] Boccia M, Piccardi L, Marco MD, Pizzamiglio L, Guariglia C (2016). Does field independence predict visuo-spatial abilities underpinning human navigation? Behavioural evidence. Experimental Brain Research.

[CR12] Bouchard S, St-Jacques J, Robillard G, Renaud P (2008). Anxiety increases the feeling of presence in virtual reality. Presence: Teleoperators and Virtual Environments.

[CR13] Brandt T, Dichgans J, Koenig E (1972). Perception of self-rotation (circular vection) induced by optokinetic stimuli. Pflügers Archiv: European Journal of Physiology.

[CR14] Brandt, T., Dichgans, J., & Koenig, E. (1973). Differential effects of central versus peripheral vision on egocentric and exocentric motion perception. *Experimental Brain Research*, *16*(5). 10.1007/BF00234474 10.1007/BF00234474 4695777

[CR15] Bremmer F, Klam F, Duhamel JR, Ben Hamed S, Graf W (2002). Visual-vestibular interactive responses in the macaque ventral intraparietal area (VIP). European Journal of Neuroscience.

[CR16] Chen A, DeAngelis GC, Angelaki DE (2011). Convergence of vestibular and visual self-motion signals in an area of the posterior sylvian fissure. Journal of Neuroscience.

[CR17] Crowne DP, Marlowe D (2011). Marlowe-Crowne Social Desirability Scale [Data set].

[CR18] Darlington CL, Smith PF (1998). Further evidence for gender differences in circularvection. Journal of Vestibular Research.

[CR19] Edwards, A. L. (1957). *The social desirability variable in personality assessment and research* (pp. viii, 108). Hinsdale: Dryden.

[CR20] Flanagan MB, May JG, Dobie TG (2002). Optokinetic nystagmus, vection, and motion sickness. Aviation, Space, and Environmental Medicine.

[CR21] Flanagan MB, May JG, Dobie TG (2005). Sex differences in tolerance to visually-induced motion sickness. Aviation, Space, and Environmental Medicine.

[CR22] Gu Y, Cheng Z, Yang L, Deangelis GC, Angelaki DE (2016). Multisensory convergence of visual and vestibular heading cues in the pursuit area of the frontal eye field. Cerebral Cortex.

[CR23] Heeter C (1992). Being there: The subjective experience of presence. Presence: Teleoper. Virtual Environ..

[CR24] Hemmerich WA, Shahal A, Hecht H (2019). Predictors of visually induced motion sickness in women. Displays.

[CR25] Hendrix, C., & Barfield, W. (1995). Presence in virtual environments as a function of visual and auditory cues. *Proceedings Virtual Reality Annual International Symposium ’95*, 74–82. 10.1109/VRAIS.1995.512482

[CR26] Hettinger LJ, Schmidt T, Jones DL, Keshavarz B, Hale KS, Stanney KM (2014). Illusory self-motion in virtual environments. *Handbook of virtual environments: Design, implementation, and applications*.

[CR27] Jáuregui Renaud K (2015). Vestibular function and depersonalization/derealization symptoms. Multisensory Research.

[CR28] Kennedy RS, Hettinger LJ, Harm DL, Ordy JM, Dunlap WP (1996). Psychophysical scaling of circular vection (CV) produced by optokinetic (OKN) motion: Individual differences and effects of practice. Journal of Vestibular Research: Equilibrium & Orientation.

[CR29] Keshavarz, B., Campos, J. L., & Berti, S. (2015). Vection lies in the brain of the beholder: EEG parameters as an objective measurement of vection. *Frontiers in Psychology*, *6*. 10.3389/fpsyg.2015.01581 10.3389/fpsyg.2015.01581 PMC4602099 26528226

[CR30] Keshavarz, B., & Hecht, H. (2011). Validating an efficient method to quantify motion sickness. *Human Factors: The Journal of the Human Factors and Ergonomics Society*, *53*(4), 415–426. 10.1177/0018720811403736 10.1177/0018720811403736 21901938

[CR31] Keshavarz B, Hettinger LJ, Vena D, Campos JL (2014). Combined effects of auditory and visual cues on the perception of vection. Experimental Brain Research.

[CR32] Keshavarz, B., Philipp-Muller, A. E., Hemmerich, W., Riecke, B. E., & Campos, J. L. (2019). The effect of visual motion stimulus characteristics on vection and visually induced motion sickness. *Displays*. 10.1016/j.displa.2018.07.005

[CR33] Keshavarz, B., Riecke, B. E., Hettinger, L. J., & Campos, J. L. (2015). Vection and visually induced motion sickness: How are they related? *Frontiers in Psychology*, *6*. 10.3389/fpsyg.2015.00472 10.3389/fpsyg.2015.00472 PMC4403286 25941509

[CR34] Keshavarz B, Speck M, Haycock B, Berti S (2017). Effect of different display types on vection and its interaction with motion direction and field dependence. I-Perception.

[CR35] Kleinschmidt A, Thilo KV, Buchel C, Gresty MA, Bronstein AM, Frackowiak RS (2002). Neural correlates of visual-motion perception as object- or self-motion. NeuroImage.

[CR36] Klosterhalfen S, Pan F, Kellermann S, Enck P (2006). Gender and race as determinants of nausea induced by circular vection. Gender Medicine.

[CR37] Lopez C, Elzière M (2018). Out-of-body experience in vestibular disorders–—A prospective study of 210 patients with dizziness. Cortex.

[CR38] Lubeck AJA, Bos JE, Stins JF (2015). Interaction between depth order and density affects vection and postural sway. PLOS ONE.

[CR39] Mach, E. (1875). *Grundlinien der Lehre von den Bewegungsempfindungen*. Leipzig: Engelmann. Retrieved from http://archive.org/details/grundlinienderle00machuoft

[CR40] Mayer-Gross W (1935). On depersonalization. British Journal of Medical Psychology.

[CR41] Montana, J. I., Tuena, C., Serino, S., Cipresso, P., & Riva, G. (2019). Neurorehabilitation of spatial memory using virtual environments: A systematic review. *Journal of Clinical Medicine*, *8*(10). 10.3390/jcm8101516 10.3390/jcm8101516 PMC6833109 31547137

[CR42] Mursic RA, Riecke BE, Apthorp D, Palmisano S (2017). The Shepard-Risset glissando: Music that moves you. Experimental Brain Research.

[CR43] Palmisano S, Allison RS, Schira MM, Barry RJ (2015). Future challenges for vection research: Definitions, functional significance, measures, and neural bases. Perception Science.

[CR44] Palmisano, S., & Chan, A. Y. C. (2004). Jitter and size effects on vection are immune to experimental instructions and demands. *Perception*, *33*(8), 987–1000. 10.1068/p5242 10.1068/p5242 15521696

[CR45] Phillips ML, Medford N, Senior C, Bullmore ET, Suckling J, Brammer MJ, David AS (2001). Depersonalization disorder: Thinking without feeling. Psychiatry Research: Neuroimaging.

[CR46] Prothero JD (1998). *The role of rest frames in vection, presence and motion sickness*.

[CR47] Riecke BE, Feuereissen D, Rieser JJ (2009). Auditory self-motion simulation is facilitated by haptic and vibrational cues suggesting the possibility of actual motion. ACM Transactions on Applied Perception (TAP).

[CR48] Riecke BE, Schulte-Pelkum J, Avraamides MN, Heyde MVD, Bülthoff HH (2006). Cognitive factors can influence self-motion perception (vection) in virtual reality. ACM Transactions on Applied Perception.

[CR49] Sasaki K, Seno T, Yamada Y, Miura K (2012). Emotional sounds influence vertical vection. Perception.

[CR50] Seno T (2013). Social inhibition of vection. Psychology.

[CR51] Seno T, Abe K, Kiyokawa S (2013). Wearing heavy iron clogs can inhibit vection. Multisensory Research.

[CR52] Seno T, Kawabe T, Ito H, Sunaga S (2013). Vection modulates emotional valence of autobiographical episodic memories. Cognition.

[CR53] Seno, T., Sawai, K., Kanaya, H., Wakebe, T., Ogawa, M., Fujii, Y., & Palmisano, S. (2017). The oscillating potential model of visually induced vection. *I-Perception*, *8*(6). 10.1177/2041669517742176 10.1177/2041669517742176 PMC5703118 29204263

[CR54] Seno, T., Taya, S., Yamada, Y., Ihaya, K., Ito, H., & Sunaga, S. (2012). Vection (self-motion perception) alters cognitive states, cognition of time, mental number line and personality. *Proceedings of the Annual Meeting of the Cognitive Science Society*, *34*(34). Retrieved from https://escholarship.org/uc/item/1cm509gc

[CR55] Seno T, Yamada Y, Ihaya K (2011). Narcissistic people cannot be moved easily by visual stimulation. Perception.

[CR56] Sierra M, Berrios GE (1998). Depersonalization: Neurobiological perspectives. Biological Psychiatry.

[CR57] Sierra M, Berrios GE (2000). The Cambridge Depersonalisation Scale: A new instrument for the measurement of depersonalisation. Psychiatry Research.

[CR58] Sierra M, Medford N, Wyatt G, David AS (2012). Depersonalization disorder and anxiety: A special relationship?. Psychiatry Research.

[CR59] So RH, Lo WT, Ho AT (2001). Effects of navigation speed on motion sickness caused by an immersive virtual environment. Human Factors.

[CR60] Spielberger CD, Sydeman SJ, Maruish ME (1994). State-Trait Anxiety Inventory and State-Trait Anger Expression Inventory. *The use of psychological testing for treatment planning and outcome assessment*.

[CR61] Weech, S., Kenny, S., Calderon, C. M., & Barnett-Cowan, M. (2020). Limits of subjective and objective vection for ultra-high frame rate visual displays. *BioRxiv*, 2020.03.19.998591. 10.1101/2020.03.19.998591

[CR62] Witkin HA, Asch SE (1948). Studies in space orientation: IV. Further experiments on perception of the upright with displaced visual fields. Journal of Experimental Psychology.

[CR63] Witkin HA, Goodenough DR (1977). Field dependence and interpersonal behavior. Psychological Bulletin.

[CR64] Wright, W. G. (2009). Linear vection in virtual environments can be strengthened by discordant inertial input. *Engineering in Medicine and Biology Society, 2009. EMBC 2009. Annual International Conference of the IEEE* (pp. 1157–1160). Retrieved from http://ieeexplore.ieee.org/xpls/abs_all.jsp?arnumber=5333425 10.1109/IEMBS.2009.5333425 19963991

[CR65] Yen Pik Sang F (2006). Depersonalisation/derealisation symptoms in vestibular disease. Journal of Neurology, Neurosurgery & Psychiatry.

